# Osteonecrosis of the Jaw Associated with Bisphosphonates Infusion for Treatment of Plasma Cell Myeloma—A Retrospective Observational Study of Northern Portuguese Population

**DOI:** 10.3390/jcm13092679

**Published:** 2024-05-02

**Authors:** Sara Sousa Ferreira, José Barbas do Amaral, José Júlio Pacheco, Filomena Salazar, Luís Monteiro

**Affiliations:** 1 UNIPRO, Unidade de Investigação de Patologia e Reabilitação Oral, Instituto Universitário de Ciências da Saúde do Norte (IUCS-CESPU), 4585-116 Gandra, Portugal; julio.pacheco@iucs.cespu.pt (J.J.P.); filomena.salazar@cespu.pt (F.S.); luis.monteiro@iucs.cespu.pt (L.M.); 2 Oral Medicine and Oral Surgery Department, Instituto Universitário de Ciências da Saúde do Norte (IUCS-N), 4585-116 Gandra, Portugal; barbas.amaral@iucs.cespu.pt

**Keywords:** osteonecrosis, jaw, bisphosphonates, risk factors, multiple myeloma

## Abstract

**Objectives**: To verify medication-related osteonecrosis of the jaw (MRONJ) frequency among patients with plasma cell myeloma (PCM) that had been treated with bisphosphonates, to identify predisposing factors that could influence the development of osteonecrosis. **Methods**: This observational retrospective study was performed at the Department of Hematology of Hospital Center of Porto (CHUP), Portugal. **Results**: The study population (n = 112) had a 15.2% (n = 17) prevalence of osteonecrosis. Clinically, bone exposure was the most frequently observed sign, present in 100% (n = 17) of the patients, followed by inflammation in 82.4% (n = 14), orofacial pain in 70.6% (n = 12), suppuration in 47.1% (n = 8), and intra or extra-oral fistula in 17.6% (n = 3) of the cases. The most frequent triggering local factor was dental extraction (82.4%). There was a dependence between the presence of extractions and the development of MRONJ (*p* < 0.001) but not with the time elapsed from the initiation of infusions with BPs and dental extractions (*p* = 0.499). In the sample of patients with multiple myeloma (MM), 13.8% were found to be more likely to develop MRONJ after an extraction. **Conclusions**: The most common local predisposing factor was dental extraction. No dependence was observed between the development of osteonecrosis and the time elapsed from the beginning of treatment with bisphosphonates infusions to surgical procedures.

## 1. Introduction

Multiple myeloma (MM), nowadays designated plasma cell myeloma (PCM), occurs in about 1% of all neoplasms and 10% of all hematological neoplasms [1]. Its incidence in Europe is around 5.6/100,000 per year [2]. In Portugal, about 300 new cases of PCM are diagnosed per year [3].

Plasma cell myeloma is characterized by a neoplastic proliferation of plasma cells (PC) in the bone marrow (BM) [1], and it may be preceded by an asymptomatic premalignant stage denominated monoclonal gammopathy of undetermined significance (MGUS), which is present in about 3–4% of the population over 50 years of age. Nearly 80% of PCM originate in non-IgM immunoglobulins, and 20% in light chain immunoglobulins [4,5,6,7,8]. The rate of MGUS progression into PCM is 0.5–1% per year, but the precise risk is influenced by the concentration and type of the monoclonal protein, by the light chain serum ratio, the BM plasmacytosis, the proportion of phenotypically abnormal PCs, and the presence of immunoparesia [8,9,10,11,12].

In patients with PCM, the malignant PCs proliferates in the BM, frequently invading the adjacent bone, causing skeletal destruction due to the increase in the osteoclastic activity and the reduction of osteoblastic functions, which results in fractures and bone pain [1,13,14]. Bisphosphonates (BPs) are specific inhibitors of osteoclastic activity. Therefore, they are used as supportive therapy to inhibit the progression of osteoclastic activity and decrease the secondary skeletal morbidity and mortality derived from this process [15]. Nonetheless, BPs are associated with a serious adverse complication: osteonecrosis of the jaw; formerly referred to as bisphosphonates-related osteonecrosis of the jaw (BRONJ) [16].

The first clinical descriptions of BRONJ were reported in 2003 and 2004 by Marx e Ruggiero [17,18]. Currently, the American Association of Oral and Maxillofacial Surgeons (AAOMS) recommends the alteration of this nomenclature to Medication-Related Osteonecrosis of the Jaw (MRONJ). In such condition, the bone becomes friable, nonviable, and eventually, exposed [16,19]. For diagnosing MRONJ, there must be a current or previous treatment with antireabsorptive or antiangiogenic agents and bone exposure, or a bone that can be probed through an intra- or extra-oral fistula in the maxillofacial region that persists for more than 8 weeks, with no history of radiotherapy with maxillary irradiation or evident mandibular bone metastases. It is important to know that patients at risk for or suffering from MRONJ can also present other common clinical conditions that should not be mistaken for MRONJ. Such conditions can be but are not limited to alveolar osteitis, sinusitis, gingivitis and periodontitis, cavities, periapical pathology, odontalgia, atypical neuralgia, fibro-osseous lesions, sarcoma, chronic sclerosing osteomyelitis and temporomandibular joint pathology. In addition, it is important to remember that exposed bones or abductions may occur in patients not exposed to anti-reabsorptive or antiangiogenic agents [19].

The scientific medical literature refers to risk factors for osteonecrosis. These risk factors include medication-dependent factors, the most important being the time of treatment with bisphosphonates [19]. Local factors such as dental extractions, periodontal problems and the use of implants or dental prostheses associated with the development of MRONJ [20].

Oral complications resulting from this condition can have a negative impact on the quality of life, affecting feeding, speech and maintenance of oral hygiene [16].

The general objective of this work was to study a homogeneous population of PCM patients with a similar BP infusion protocol. Specifically, we aimed to verify the frequency of MRONJ, to identify the main oral signs and symptoms, as well as the local triggering factors and associated comorbidities, and to evaluate how the time elapsed between the beginning of BPs and the performance of surgical procedures, such as dental extractions, influence the development of osteonecrosis.

## 2. Materials and Methods

This retrospective observational study was performed at the Service of Clinical Hematology of the Hospital de Santo António (HSA)—Centro Hospitalar Universitário do Porto (CHUP), Porto, Portugal, between April and June 2018. This work was approved by the Ethics Committee and the Department of Education, Training and Research, and by the Board of Directors of the CHUP (Ref. 2017.249/218-DEFI/210-CES). The authors hereby confirm to have read the Helsinki Declaration, which has been followed during this investigation.

In order to carry out this study, we manually consulted paper (1997–2003) and electronic (2004–2017) clinical records of all patients diagnosed with PCM between 1997 and 2017. In total, 357 clinical records were examined. In the course of this analysis, we proceeded to registered demographic data, such as sex and age, the year of diagnosis of PCM and the consultation in the Department of Stomatology and Maxillofacial Surgery. Accordingly, the study sample consisted of 112 patients who had received treatment with zoledronic acid. Of those, only the patients who had undergone surgical procedures, such as dental extractions, were considered for analysis. Finally, patients who developed osteonecrosis were identified, and their stage was classified according to the Ruggiero et al. 2014 update [19].

We also analyzed the signs and symptoms observed in the Stomatology and Maxillofacial Surgery consultation such as pain, inflammation, suppuration, fistula and bone exposure, the presence of ill-fitted dentures and the presence of preexisting inflammatory dental diseases.

The presence of comorbidities such as anemia (Hg < 10 g/dL), high blood pressure, diabetes, renal failure, and smoking habits were also verified. Finally, PCM was classified according to the type and stage of the tumor.

### 2.1. Statistical Analyses

Statistical analysis of the results was carried out using the SPSS software version 24. Logistic regressions (Binary Logistic Regression) were performed to evaluate the dependence between gender, age and dental extractions and between the time elapsed between the initiation of BPs infusions and the extractions, in the development of MRONJ. The value of statistical significance was set at *p* < 0.05.

### 2.2. Selection Criteria

During the discussion of results, bibliographic research was carried out using the databases NICE—The National Institute for Health and Care Excellence, The Cochrane Collaboration, DARE—Database of Abstracts of Reviews of Effects, and MedLine, accessed via PubMed. The keywords used for research were “osteonecrosis” and “jaw”, “osteonecrosis” and “bisphosphonates”, “osteonecrosis” and “risk factors”, “osteonecrosis” and “multiple myeloma”, “multiple myeloma” and “bisphosphonates”.

The scientific manuscripts included in this study were chosen according to the following selection criteria: (1) date of publication between 2003 and 2018; (2) written in English; (3) and whose title contained the keywords used as inclusion criteria. Manuscripts with little relevant content were excluded. Correspondingly, a total of 17,099 articles were obtained at an initial stage. Of those, 37 were selected. Finally, articles that were identified using manual search were added to the list of the selected articles. At the end of the research, 56 articles were obtained.

## 3. Results

The sample was composed of 112 patients, of which 61 were female (54.5%) and 51 male (45.5%), mean age of 72 ± 12 years. The treatment protocols used in all cases of the sample were very similar, i.e., monthly 4 mg of BP (zoledronic acid) infusions over a period of 24 months. In this study population, the frequency of osteonecrosis observed was 15.2%. The mean age of patients with MRONJ was 70 ± 12 years. The time elapsed between the initiation of bisphosphonates infusions and the development of osteonecrosis was 28.2 ± 23.4 months.

**Figure 1:**
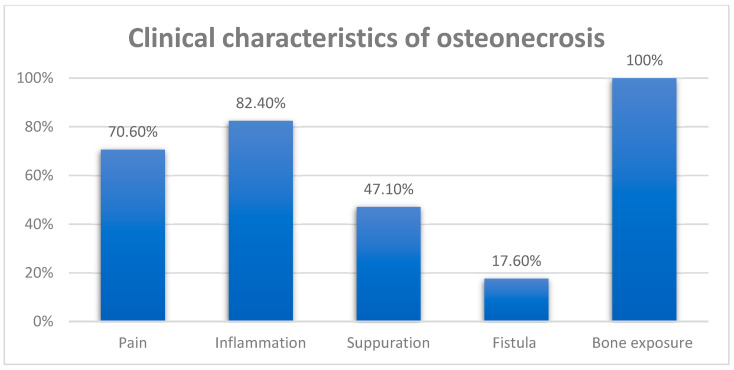
Clinical signs and symptoms of osteonecrosis.

The most frequent clinical characteristic found was bone exposure, observed in 100% of the cases. Figure 1 shows the prevalence of clinical signs and symptoms observed in the 17 patients with PCM who developed osteonecrosis of the jaw. Osteonecrosis was most frequently found in the mandible (64.7%).

The most frequent triggering local factor was dental extraction, which was observed in fourteen patients (82.4%). It is noteworthy that, although this was the main triggering factor, three patients (17.6%) also presented oral inflammatory diseases, and three (17.6%) had a dental prosthesis. There were three cases (17.6%) in which bone exposure occurred spontaneously or without apparent cause.

As for the presence of comorbidities, four cases (23.5%) presented Hg values of <10 g/dL; three cases (17.6%) had diabetes; and six cases (35.3%) had high blood pressure and renal failure. The presence of smoking habits was also verified in two (11.8%) cases.

Table 1 shows the demographic characteristics, such as sex and age, myeloma type and stage, comorbidities, local triggers and site of osteonecrosis, and the MRONJ stage of the seventeen patients who developed MRONJ, according to the Ruggiero et al. 2014 update [19].

Using a binary logistic regression, we analyzed the relation of some variables and the occurrence of osteonecrosis. There were no significant relations between gender (*p* = 0.891) and age (*p* = 0.657) with the occurrence of MRONJ. However, it was verified that there is a dependence between the presence of extractions and the development of MRONJ (*p* < 0.001; Exp(B) = 13.806, 95% CI of 3.651–52.202). That is, patients who received treatment with zoledronic acid infusions and undergo this type of procedure are 13.8% more likely to develop osteonecrosis of the jaw than patients who do not. Moreover, when we evaluate the number of extractions, patients with multiple extractions are 17.8% more likely to develop osteonecrosis compared to patients that did not perform any extraction (*p* < 0.001; Exp(B) = 17.750, 95% CI of 4.194–75.124). On the other hand, no dependence was observed between the development of osteonecrosis and the time elapsed from the initiation of infusions with BPs to the performance of dental extractions (*p*= 0.499; Exp(B) = 1.010, 95% CI of 0.982–1.038).

## 4. Discussion

Epidemiological data on the prevalence and incidence of osteonecrosis of the jaw (ONJ) are limited and usually not based on prospective studies or population surveys [21]. The prevalence of osteonecrosis induced by infusion of BPs found in our 112 PCM patients’ sample was 15.2%. This is in accordance with data found in the literature on the osteonecrosis prevalence in PCM, which range from 0% to 20.5% (median value of 5.1%) [22]. The development of ONJ is directly related to the use of antireabsorptive agents [23,24,25] and significantly correlated with the number of applied doses [26,27,28]. In fact, in Auzina et al.’s sample it was shown that most patients who developed MRONJ received an IV treatment period of more than 1 year [29]. Consequently, variations in these factors as well as differences in the adopted dosage protocols may be the reason for the discrepancies observed in the prevalence de ONJ [22].

There are anatomical factors associated with the development of MRONJ [19]. Bisphosphonates inhibit the differentiation and function of the osteoclasts and increase their apoptosis, leading to decreased bone reabsorption and bone remodeling [30,31,32]. The increased remodeling rate found in the maxilla and the jawbones may explain the differential predisposition to the jawbones concerning other bones of the axial or appendicular skeleton [19]. Additionally, there is a higher predisposition to developing MRONJ in the mandible (64.7%) in comparison with the maxilla (35.3%), but the condition can occur in both bones simultaneously [33,34,35,36,37,38].

The development of MRONJ in oncologic patients is associated with local, demographic, and systemic risk factors, as well as with factors related to other types of drugs, and with genetic factors [19]. The relevant risk factors for the development of ONJ that have been reported in the literature are the dose of BPs [28,34,37,39,40,41], the duration of exposure [25,27,29,35,40], the type of BPs [25,27,40,42] with zoledronic acid potentiating osteonecrosis development the most [25,28,40,43,44], the use of oral BPs [25], dental extractions [29,33,35,40,43,45], inflammatory dental disease [34,46], the use of dental prosthesis [33,43,45,47], local suppuration [25], treatment with corticoids [20,35,36,47], erythropoietin [20,35], cyclophosphamide [35] and angiogenic inhibitors [20], diabetes [20,27,47], osteoporosis [40], anemia [35], hypothyroidism [20,27], renal dialysis [35], smoking [20,27,47,48], age [33,35,47,49] and sex [33,47].

Although there are no studies in the literature reporting on an association between high blood pressure and the development of osteonecrosis, in this study population, 35.3% of MRONJ patients had high blood pressure. This may be associated with the high prevalence of high blood pressure in Portugal. It is estimated that about 36% of the Portuguese population has high blood pressure [50]. Likewise, the percentage of patients with diabetes and MRONJ may be a reflection of the prevalence of diabetes in the Portuguese population, corresponding to around 13.3% [51].

In this study, we chose not to test the duration of treatment as an independent risk factor since the objective was to detect the prognostic factors to avoid MRONJ and, not to propose as a preventive measure, a reduction in the number of infusions with zoledronic acid. However, other authors reported that longer treatment with zoledronic acid is associated with a greater risk for the development of MRONJ [27,37,48].

Although in the present study we did not find statistically significant differences between males and females, the higher prevalence of this complication in the female population reported in the literature may be an underlying reflection of diseases such as osteoporosis and breast cancer, for which some types of BPs are prescribed [19].

According to the literature, patients over 65 years of age have a greater predisposition to MRONJ [28,33]. The patients of our sample who developed MRONJ had, on average, 70 ± 11 years (range: 44 to 85 years). In fact, with age, the regenerative processes decrease. Besides, elderly patients have a higher risk of complications such as cavities, periodontal problems that can potentiate the need of surgery and the need to wear dental prosthesis; all of which are risk factors for the development of MRONJ. On the other hand, advanced age may reflect a longer course of illness and, consequently, a longer time of exposure to BPs [28]. Thus, further studies are needed to assess age as an independent risk factor.

Dentoalveolar surgery is a major local risk factor for the development of MRONJ [19,29]. Several studies conducted on patients with MRONJ who underwent dental extractions show that in 54% to 69% of the cases this was the main factor triggering osteonecrosis of the jaw [23,33,36,39]. In fact, 82.4% of the patients in this sample who had MRONJ had undergone one or more dental extractions. It is estimated that in cancer patients exposed to bisphosphonates IV, the risk of developing MRONJ after undergoing dental extraction varies between 1.6 and 14.8% [19]. In the present sample, in patients with PCM exposed to zoledronic acid, the probability of developing osteonecrosis of the jaw after undergoing dental extraction is 13.8%. Undergoing dental extraction and, consequently, suffering a bone and mucosa injury, exposes free tissue to the oral microflora. Aggregates of polymorph nuclear leukocytes and bacteria are frequently observed in necrotic tissue, and the presence of bacterial microfilms has been associated with active osteoclast resorption on the bone surface [37,52]. The inhibitory effects of BPs on the proliferation and viability of oral keratinocytes [53,54,55] may aggravate the integrity of the oral mucosa and increase the risk of infection [21]. In addition, the large blood supply in the jaws and the rapid impact-induced bone turnover rate in dental procedures may concentrate high doses of bisphosphonates in the area [44]. However, the risk of developing ONJ in patients who undergo other types of dental procedures, such as implant placement and endodontic or periodontal procedures is unknown. Due to the lack of data in the literature, the AAOMS considers procedures that require the exposure and manipulation of at-risk bones comparable to the risk associated with dental extractions [19].

The sequence of events that cause the development of ONJ is not clear. It is not known whether necrosis precedes or follows infection [21]. Although in the present study, only three patients (17.6%) who developed ONJ had pre-existing inflammatory dental disease, such as periodontal or periapical disease, dental disease is a well-established risk factor for ONJ [34,46]. A study in 2012, which evaluated patients with cancer and MRONJ, showed that inflammatory dental diseases were a risk factor in 50% of the cases [36]. On the other hand, the administration of IV bisphosphonates could cause a greater degree of inflammation as a result of their ability to inhibit bone remodeling [56]. However, since a common treatment of inflammatory dental disease is dental extraction, pre-existing dental disease may confuse the relationship between dental extraction and the risk for MRONJ. Thus, it would be important to study the association between dental extraction and MRONJ, verifying the preexistence or not of inflammatory dental disease [19].

On the other hand, the use of dental prosthesis in dental patients with cancer exposed to zoledronate has also been associated with increased risk of MRONJ [33,43,45,47]. Vahtsevanos et al. analyzed a sample of 1621 cancer patients treated with zoledronate, ibandronate or pamidronate IV, and found a two-fold higher risk in patients with dental prosthesis [43]. The use of dental prostheses, especially if poorly adapted, can cause injuries and weaken the mechanical barrier of the oral mucosa, allowing the oral microflora to enter the bone [45]. In the present sample, we observed three patients (17.6%) with a poorly adapted dental prosthesis.

The use of corticosteroids is also a risk factor for MRONJ [35,36,47]. In this research, it was not possible to study the relationship between the use of corticosteroids and the development of MRONJ since all patients were treated with similar protocols. However, it is possible that the immunosuppressive effects of corticosteroids delay healing and alter the oral microflora, thus increasing the risk of infection and MRONJ [36].

Although in this study it was not possible to investigate which patients were taking antiangiogenic agents, drugs such as thalidomide are commonly prescribed to patients with MM. Angiogenesis is a process that involves the growth, migration and differentiation of endothelial cells to form new blood vessels, positively influencing tumor growth and invasion of the vessels, resulting in tumor metastasis. Angiogenesis requires the binding of signaling molecules, such as vascular endothelial growth factor (VEGF), to endothelial cell receptors. Such signaling promotes the growth of new blood vessels. Inhibitors of angiogenesis interfere with the formation of new blood vessels through binding to signaling molecules, thereby disrupting the angiogenesis signaling cascade [19]. Osteonecrosis is classically considered an interruption in vascular supply or avascular necrosis. Therefore, it is not surprising that the inhibition of angiogenesis is one of the main hypotheses in the pathophysiology of ONJ [55,57,58].

Regarding smoking, although it has been consistently associated with the development of MRONJ, the literature is not consensual. In 2009, in a case–control study, smoking came close to a statistically significant value as a risk factor for ONJ in cancer patients [45]. More recently, in a study in zoledronate-treated patients, smoking was not associated with the development of ONJ [46]. Similarly, the study by Vahtsevanos et al. showed no relationship between smoking and the development of ONJ [43]. Although only two (11.8%) patients who developed MRONJ had smoking habits, it is known that carcinogenic components of tobacco delay healing [59], aggravate periodontal disease [60] and cause epithelial changes [48]. In addition, nicotine causes an increase in vasoconstriction in the bone, leading to ischemic stages that may be behind the pathophysiology of osteonecrosis [59,61].

Bone exposure was the most observed clinical sign, present in 100% of the cases. This result may be a reflection of a late diagnosis. No patient in our sample was diagnosed with stage 0, in which there are no clinical signs of necrotic bone. However, there are nonspecific clinical findings, symptoms and radiographic changes. In this case, the symptoms that may be present are odontalgia without apparent cause, mandibular bone pain that may radiate to the temporomandibular region, sinus pain that may be associated with inflammation and thickening of the maxillary sinus wall and altered sensorineural function [19]. In this study, 70.6% of the patients presented orofacial pain. Clinically, inflammation was observed in 82.4% of the cases, 47.1% presented suppuration, followed by the presence of a fistula, present in 17.6% of patients.

### Strengths and Limitations

Although the present study takes into account only a period of twenty years (1997–2017) of MRONJ cases registered in just one Portuguese hospital, it must be emphasized that the database used for the research had to be built from scratch since nothing similar was available in the mentioned institution or, as far as we know, in the country. The resulting database is now available for other research, and it can be an important help for the comprehension of lesions in this type of patient. We also acknowledge some limitations such as the lack of detailed information of some clinical variables included in the study. Nevertheless, and following our initial objectives we include strong and feasible variables information to avoid the presence of bias related with retrospective studies. To our knowledge, these data are the first to document the frequency of MRONJ associated with bisphosphonates infusion for the treatment of plasma cell myeloma in an observational study in Portugal.

## 5. Conclusions

Osteonecrosis of the jaw is a relatively frequent complication in patients with PCM who undergo treatment with zoledronic acid intravenous. The most predisposing local factor is dental extraction. In our sample of patients with PCM, it was found that 13.8% were more likely to develop MRONJ after extraction, and no dependence was observed between the development of osteonecrosis and the time elapsed from the initiation of BP infusions to the performance of surgical procedures. Thus, to avoid such procedures, all patients should be examined by a dentist and/or stomatologist before initiating therapy in order to ensure that all inflammatory dental conditions are treated before BPs are administered.

## Figures and Tables

**Table 1:** Clinical characteristics of the 17 patients with MRONJ.

Gender	Age (Years)	Ig	Stage	Comorbidities					Osteonecrosis	MRONJ Stage	Time Elapsed between the Infusion of BPs and the Development of Osteonecrosis (Months)	Triggering Local Factors
				Values Hg (g/dL)	Diabetes	HBP	Smoking	Kidney Failure				
M	72	IgG k			Yes	No	No	Yes	Maxilla	1	73	Extraction
M	66	IgG k	II	11.8	No	No	No	No	Jaw	2	22	Extraction
F	75	IgG k	II	10.9	No	Yes	No	No	Maxilla	2	11	Extraction + Inflammatory Oral Disease
M	84	IgG k	III	12.9	No	No	No	Yes	Jaw	1	32	Extraction + Dental prosthesis
M	62	IgG L			No	No	No	Yes	Maxilla	2	60	Extraction + Inflammatory Oral Disease
F	80	IgG L	II	8.9	Yes	Yes	No	Yes	Jaw	2	24	Spontaneous
M	73	IgG k	II	8.2	No	No	No	No	Jaw	2	2	Spontaneous
F	79	IgG k		11.8	No	No	No	Sim	Jaw	3	25	Extraction + Dental prosthesis
F	79	IgG L	I		No	Yes	No	No	Jaw	2	41	Extraction + Dental prosthesis
M	85	IgA L	I	12.8	Yes	Yes	No	No	Jaw	2	30	Extraction
M	61	IgG k	II	10.6	No	No	Yes	Yes	Maxilla	3	33	Extraction + Inflammatory Oral Disease
F	44	IgG L	II	12.2	No	No	No	No	Jaw	2	77	Extraction
F	75	IgA L	II	12.2	Yes	Yes	No	No	Maxilla	1	19	Spontaneous
F	84	IgA L	II	11.4	No	Yes	No	No	Jaw	2	2	Extraction
F	57	Light chain k	I	12.4	No	No	Yes	No	Jaw	1	4	Extraction
M	61	IgG k	I	9.2	No	No	No	No	Jaw	2	2	Extraction
F	59	IgA k	III	9.1	No	No	No	No	Maxilla	2	21	Extraction

Abbreviations: Ig, immunoglobulin; HBP, high blood pressure; Hg, hemoglobin; MRONJ, medication-related osteonecrosis of the jaw.

## Data Availability

The original contributions presented in the study are included in the article.
